# Case Report: Isolated Epileptic Seizures Associated With Anti-LGI1 Antibodies in a 7-Year-Old Girl With Literature Review

**DOI:** 10.3389/fped.2022.856775

**Published:** 2022-05-31

**Authors:** Ying Wang, Wei-Hua Zhang, Yuan Wang

**Affiliations:** ^1^Department of Neurology, Henan Children’s Hospital, Zhengzhou Children’s Hospital, Children’s Hospital Affiliated to Zhengzhou University, Zhengzhou, China; ^2^National Centre for Children’s Health, Beijing Children’s Hospital, Capital Medical University, Beijing, China

**Keywords:** LGI1 antibodies, isolated seizures, autoimmune encephalitis, immunotherapy, children

## Abstract

We describe the case of a 7-year-old girl with anti-leucine-rich glioma-inactivated 1 (anti-LGI1) antibodies (Abs) who presented with isolated epileptic seizures. Her refractory focal seizures did not respond to anti-seizure medicines but responded rapidly to immunotherapy. She remained seizure-free at 2 years follow-up. Reviewing the literature, isolated epileptic seizures have not been reported as the phenotype of anti-LGI1 autoimmunity in children. Our study indicated that screening for anti-LGI1 Abs is necessary for children with severe and/or drug-resistant new-onset focal epileptic seizures.

## Introduction

Anti-leucine-rich glioma-inactivated 1 (anti-LGI1) encephalitis is the second most frequent type of autoimmune encephalitis in adults (1). It is often encountered in older men. Seizures occur in more than 80% of patients and are usually accompanied by other neurological symptoms including cognitive impairment, psychiatric disorders, and faciobrachial dystonic seizures (FBDS). Though rare in children, most pediatric patients were reported to present with classical limbic encephalitis. However, epilepsy has not been reported as the only manifestation in pediatric patients. Here, we describe the case of a 7-year-old girl with isolated focal epileptic seizures associated with serum LGI1 antibody positivity who responded well to immunotherapy.

## Case Presentation

A 7-year-old girl was admitted to our hospital with frequent focal seizures for fifteen days. The seizures manifested as bilateral eye deviation to the right; rhythmic clonic movements of the right facial muscles; upturned corners of the right mouth; and clawing of the right hand with right upper limb tonic movements, with or without right lower limb tonic movements lasting approximately 3–20 s, 1–2 times per day initially. The frequency gradually increased to approximately 20 times per day. The patient was normal during the interictal period. Other neurologic symptoms, such as behavioral changes, hallucinations, confusion, memory impairment, and cognitive impairment, were not observed. No treatment was administered during the first 15 days. She visited our hospital because the seizures were worsening. Physical and neurological examination results were normal upon admission. Memory, numeracy, and spatial cognition were normal. The patient was healthy with normal developmental milestones. The patient had no family history of seizures or autoimmune disorders. She participated in school activities that were appropriate for her age. She was in 1st grade at primary school and had excellent academic performance.

**FIGURE 1:**
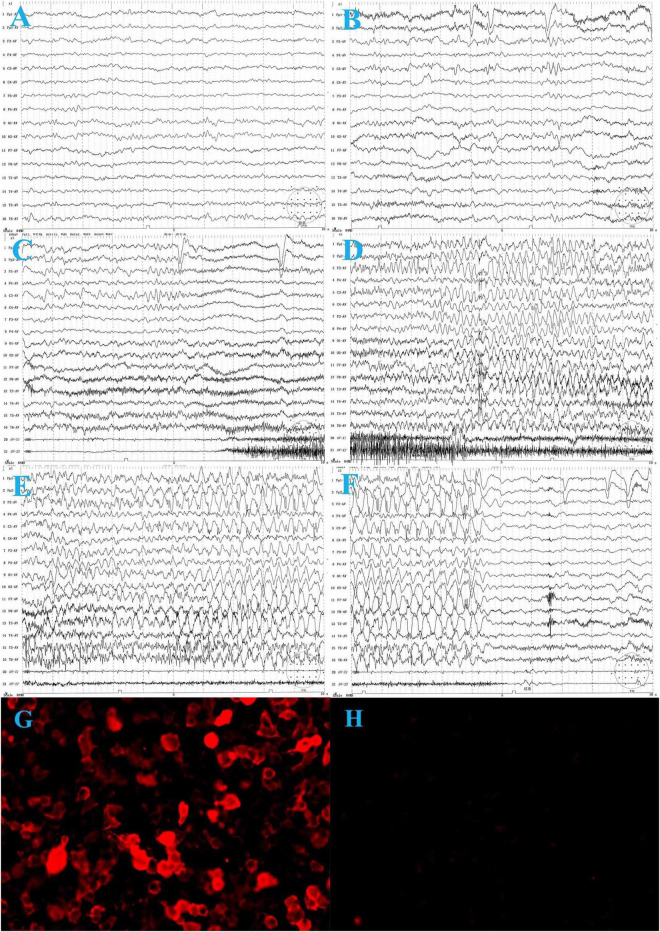
The electroencephalogram (EEG) of the patient before treatment. **(A)** Background activity: 6∼7 Hz low to medium voltage theta waves over bilateral occipital regions during wakefulness. **(B)** Interictal VEEG: spike and slow waves in the left frontal and central regions during wakefulness. **(C–F)** Ictal EEG: the focal seizure begins with low voltage, fast-wave rhythm over the left frontal and central regions, gradually progressing to spike-and-wave discharges in all regions, predominantly in the left frontal region, lasting about 35 s, during which the electromyography (EMG) burst. The seizure ends and is followed by diffuse background voltage attenuation. **(G)** Serum anti-LGI 1-Ab: 1:30. **(H)** One month after treatment, serum anti-LGI 1 Ab is negative.

On laboratory examination, complete blood count, renal and liver function tests, and thyroid function tests were normal. No hyponatremia was observed. The metabolic examination results, including serum ammonia, lactate, homocysteine, and blood urine amino acid organic acid profiles, were normal. Cranial magnetic resonance imaging (MRI) was normal. Electroencephalography (EEG) revealed slow background activity (Figure 1A) and epileptiform activity in the left frontocentral region (Figure 1B). The ictal video-EEG (VEEG) captured focal epileptic seizures originating from the left frontal and central regions (Figures 1C–F). Tumor screening for blood tumor markers (HCG, AFP, CEA, and NSE), lung computed tomography, and abdominal B-scan ultrasonography were negative. Cerebrospinal fluid (CSF) cytology results were normal and negative for oligoclonal bands. CSF glucose, chloride, protein, and lactic acid levels were within normal ranges. Serological and CSF assays for infectious agents, including viral etiologies, tuberculosis, *Mycoplasma pneumoniae*, and *Chlamydia pneumoniae*, yielded negative results. A cell-based indirect immunofluorescence antibody assay was used to detect the related antibodies (Abs). Anti-LGI1 Abs was detected at a titer of 1:30 (Figure 1G) in the serum and was negative in the CSF. Abs against NMDAR, GABABR, AMPAR, Caspr2, Amphiphysin, CV2, PNMA2 (Ma2), Hu, Ri, Yo, and anti-thyroid Abs were negative in the CSF and serum.

**FIGURE 2:**
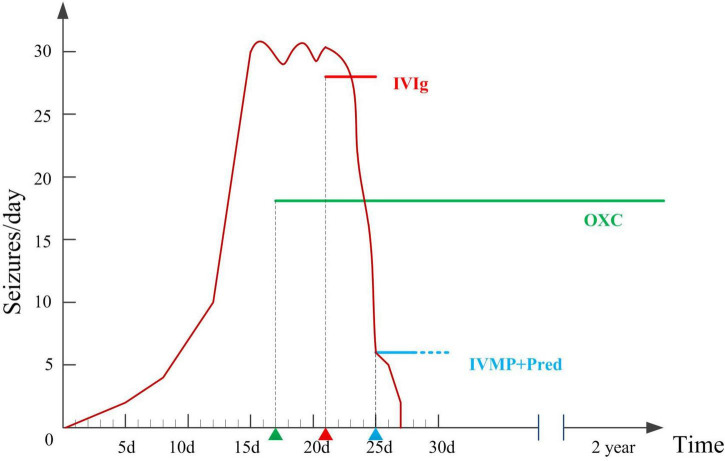
Therapeutic interventions and number of seizures per day during hospitalizations and two years follow-up.

Oxcarbazepine (OXC) was administered to control frequent seizures after admission (17th day after onset), with poor response. The etiology of the patient’s epilepsy is important for treatment. The six etiologies of epilepsy include structural, genetic, infectious, metabolic, and immune disorders, and also an unknown group. Structural etiology was excluded by the normal brain MRI. The patient was previously healthy, with normal developmental milestones, and all metabolic examinations were normal, hence metabolic etiology was excluded. She had acute onset with no family history of seizures, which did not support genetic epilepsy. Infectious etiology was excluded for the absence of infection symptoms, normal CSF test results, and infectious agents. Since she had an acute onset condition with positive anti-LGI1 antibody in serum and slow background activity in EEG, autoimmune epilepsy should be considered. Hence, we tried immunotherapy. Immunotherapy was initiated on the 21st day after onset. Intravenous immunoglobulin (0.4 g/d/kg body weight, 5 days) was administered, and the number of seizures significantly decreased. On the 25th day after onset, high-dose intravenous methylprednisolone (20 mg/kg) was administered once a day for 3 days, followed by oral prednisolone treatment for 6 months. The seizure frequency gradually decreased 2 days after intravenous methylprednisolone, and the seizures disappeared. One month later, serum anti-LGI1 Abs were negative (Figure 1H). The slow waves of the VEEG improved. The patient was seizure-free at the last follow-up (2 years). She was as healthy as before, without neurological dysfunction. She returned to school soon after she was discharged from the hospital, and everyone around her thought her performance was the same as that before she was ill.

## Discussion

Antibodies directed against LGI1 were discovered in 2010. Anti-LGI Abs are recognized as one of the anti-cell surface Abs associated with autoimmune encephalitis. Their presence is associated with characteristic symptoms such as faciobrachial dystonic seizures (FBDS), temporal lobe seizures, memory dysfunction, hyponatremia, and neuropsychiatric symptoms. Association with tumor occurs in up to 11–13% of cases with anti-LGI1 Abs, which has been mostly reported in adults. More than 80% of the adult patients with anti-LGI1 encephalitis had epileptic seizures. Focal epileptic seizures were also prominent. Most patients had frequent seizures and responded poorly to anti-seizure medicines. However, Status epilepticus (SE) is rare.

Recently, only a small number of children with this condition have been reported. To date, there has not been a well-defined description of the clinical spectrum in children. Encephalitis is one of the most common clinical symptoms of pediatric LGI1 autoimmunity (2). With the limitations imposed by the low number of cases, differences in published adult cohorts include the absence of faciobrachial dystonic seizures, hyponatremia, and tumors. Seizures, as an accompanying symptom, were present in 57.1% of LGI1-positive pediatric patients. However, isolated epileptic seizures without any other neurological symptoms were very rare in adults and had not been reported in cases of LGI1 autoimmunity in children. We reviewed 12 well-documented pediatric patients (Table 1) in the literature (3–8). Seven patients had seizures, but unlike our patient, all of them were accompanied by other neurological symptoms such as encephalopathy, sleep disturbances, dysfunction of recent memory, or behavioral changes. The rate of reported seizures in children is lower than in adults. The primary form is focal seizures with no status epilepticus, which is similar to that in adults.

**TABLE 1:** Clinical characteristics and treatment response in pediatric patients seropositive for leucine-rich glioma-inactivated 1 (LGI1)-IgG.

Patient, No.	Age at Onset, y	Sex	Encephalopathy	Seizure/Type	Sleep Disturbances	FBDs	PNS	ANS	Neuropsychiatric/Behavioral	CSF			Brain MRI	EEG	Treament		MRS	Outcome	Follow-up Period, mo
										cells	protein (g/L)	OCB			Immunotherapy	ASM			
1	9	M	+	+/focal	+	−	−	−	+	N	N	N	+	+	IVIg	−	1	improved	7
2	17	F	+	+/focal	+	−	+	+	+	N	N	+	N	+	IVIg/AZA	−	1	improved	20
3	5	F	−	+/focal	+	−	−	−	+	N	N	N	N	+	−	VPA/LCM	1	chronic epilepsy	40
4	13	M	+	+/focal	−	−	+	−	+	22	0.77	N	+	−	IVIg/IVMP	−	0	improved	16
5	8	F	+	+/partial with secondary generalized	−	−	−	−	+	N	N	N	+	+	IVIg/IVMP	LEV	NA	recovered	NA
6	14	M	+	+/focal	−	−	−	−	+	N	N	+	+	−	IVMP, PE, MMF	−	1	improved	60
7	18	M	+	+/myoclonus	−	−	−	+	+	69 → 271	0.468	N	+	−	IVIg/IVMP	−	NA	normal	9
8	17	F	+	-/-	+	−	+	+	−	N	N	N	N	NA	IVIg	−	1	improved	21
9	17	F	−	-/-	−	−	+	+	−	NA	NA	NA	NA	NA	−	−	2	NA	3
10	14	F	−	-/-	−	−	+	−	+	NA	NA	NA	N	−	−	−	1	stable	6
11	6	F	−	-/-	−	−	−	−	+	N	N	N	N	−	IVMP/IVIg	−	0	complete resolution	2
12	7	F	+	-/-	+	−	−	−	+	N	N	N	N	+	IVIg/IVMP	−	NA	good outcome	NA

*FBDS, faciobrachial dystonic seizures; PNS, peripheral nervous system; ANS, autonomic nervous system; CSF, cerebrospinal fluid; MRI, magnetic resonance imaging; EEG, electroencephalogram; ASM, anti-seizure medicine; MRS, modified Rankin Scale; F, female; M, male; NA, not available; IVIg, intravenous Ig; IVMP, intravenous methylprednisolone; PE, plasma exchange; MMF, mycophenolate mofetil; N, normal; VPA, valproic acid; LCM, lacosamide; LEV, levetiracetam; +, positive; −, negative; OCB, oligoclonal bands; AZA, azathioprine.*

Faciobrachial dystonic seizures are specific to anti-LGI1 encephalitis, although only in a minority of patients. FBDS are involuntary contractions lasting 1–2 s, are unilateral, and occur up to 100 times a day. FBDS are considered as non-epileptic episodes due to the common absence of ictal EEG abnormalities. However, the underlying mechanism of FBDS remains controversial. Some researchers found that the percentage of abnormal basal ganglia signals in patients with FBDS was significantly higher than in non-FBDS patients. Therefore, they thought that FBDS were related to the function of the basal ganglia, making them a type of movement disorder. FBDS present as high-frequency attacks (40 ± 17 times per day) with short episodes (2.1 ± 0.7 s) and are always unilateral (9), so they are easy to differentiate from generalized epilepsy. However, although the mean seizure frequency is lower and the duration of focal-onset seizures are longer than that of FBDS, it is difficult to distinguish them from focal-onset epilepsy. This group of patients was easily diagnosed with classic mesial temporal lobe epilepsy. However, symptoms such as fear, epigastric rising, staring, and automatisms were difficult to observe in pediatric patients. Thus, ictal VEEGs are needed to identify FBDS or epileptic seizures during focal episodes, especially short-term episodes. It should be emphasized that one-third of patients can simultaneously develop FBDS and epileptic seizures (10).

The attacks in our patient showed prominent unilateral motor seizures, but most lasted longer than 10 s, and the epileptic wave discharge on EEG during the episode did not display the features of typical FBDS. Few studies on FBDS in children have been conducted. Whether FBDS have different characteristics in children requires further investigation.

We describe a pediatric case of isolated focal seizures associated with anti-LGI1 Abs that responded poorly to anti-seizure medicines but responded well to immunotherapy. This phenomenon has prompted the study of the role of autoimmunity in epileptic seizures. Immunity has been reported as one of the possible etiologies of new-onset epilepsy or established epilepsy of unknown etiology (11, 12). Among adult patients with epilepsy of unknown etiology, more than 20% had autoantibodies that were strongly suggestive of autoimmune epilepsy, mainly GAD65 Abs and LGI1 Abs (12). In children, about 10% of the patients were positive for Abs, of which the top three were voltage-gated potassium channel (VGKC) complex, contactin-associated protein-like 2 (CASPR2), and N-methyl-D-aspartate receptors (NMDARs) (11). Most autoantibody-mediated epileptic seizures are of the focal type. Therefore, for patients with unknown drug-resistant new-onset severe focal epilepsy, screening for anti-neural antibodies is necessary. Prompt immunotherapy will benefit these patients.

Currently, there are several criteria for selecting and sorting patients with putative autoimmune epilepsy. One is the Antibody Prevalence in Epilepsy (APE) score proposed by Dubey et al. (12). An APE score of 4 or higher (specificity, 82.0%; sensitivity, 82.6%) can be used to select adult patients for antibody testing. APE < 4 is not sufficiently high to be classified in the putative autoimmune epilepsy category. However, because our patient had a short history and had been administered immunotherapy at the early stage of the course of the disease, some symptoms such as encephalopathy might not have appeared, and the second anti-seizure medications were not administered at this time, so the APE score was low. Therefore, further research is needed to determine whether it is necessary to adjust the evaluation criteria of APE. However, the patient did not fulfill the autoimmune encephalitis criteria introduced in 2016 (13) or the pediatric autoimmune encephalitis criteria defined in 2020 (14). Similar patients in the past may not have been tested for immune factors, so this requires further studies with large sample sizes.

Our study had several limitations. The patient was not subjected to proper cognitive tests during an acute episode or follow-up. Because the best assay technologies were not available to us at the time of our study, we could not perform assays such as end-point titer determination and tissue-based antibody assays. We will incorporate them in future cases.

In summary, epileptic seizures may be isolated manifestations of LGI1 autoimmunity in children. For pediatric patients with severe and/or drug-resistant new-onset focal epileptic seizures, routine antineuronal antibody screening is recommended to avoid delay of immunotherapy and improve prognosis.

## Data Availability Statement

The datasets for this article are not publicly available due to concerns regarding participant/patient anonymity. Requests to access the datasets should be directed to W-HZ, 15811076790@163.com; YuW, 13838393216@163.com.

## Ethics Statement

Written informed consent was obtained from the individual(s), and minor(s)’ legal guardian/next of kin, for the publication of any potentially identifiable images or data included in this article.

## Author Contributions

YiW drafted the manuscript and attended the patient. YuW did the follow-up with the patient. W-HZ and YuW edited the manuscript. All authors contributed to the article and approved the submitted version.

## Conflict of Interest

The authors declare that the research was conducted in the absence of any commercial or financial relationships that could be construed as a potential conflict of interest.

## Publisher’s Note

All claims expressed in this article are solely those of the authors and do not necessarily represent those of their affiliated organizations, or those of the publisher, the editors and the reviewers. Any product that may be evaluated in this article, or claim that may be made by its manufacturer, is not guaranteed or endorsed by the publisher.

## References

[1] van Sonderen A Petit-Pedrol M Dalmau J Titulaer MJ. The value of LGI1, Caspr2 and voltage-gated potassium channel antibodies in encephalitis. *Nat Rev Neurol.* (2017) 13:290–301. 10.1038/nrneurol.2017.43 28418022

[2] Nosadini M Toldo I Tascini B Bien CG Parmeggiani L De Gaspari P LGI1 and CASPR2 autoimmunity in children: systematic literature review and report of a young girl with Morvan syndrome. *J Neuroimmunol.* (2019) 335:577008. 10.1016/j.jneuroim.2019.577008 31352183

[3] López-Chiriboga AS Klein C Zekeridou A McKeon A Dubey D Flanagan EP LGI1 and CASPR2 neurological autoimmunity in children. *Ann Neurol.* (2018) 84:473–80. 10.1002/ana.25310 30076629

[4] Erer Özbek S Yapıcı Z Tüzün E Giriş M Duran S Taşkapılıoğlu Ö A case of hyperkinetic movement disorder associated with LGI1 antibodies. *Turk J Pediatr.* (2015) 57:514–7. 27411421

[5] AlHakeem AS Mekki MS AlShahwan SM Tabarki BM. Acute psychosis in children: do not miss immune-mediated causes. *Neurosciences (Riyadh).* (2016) 21:252–5. 10.17712/nsj.2016.3.20150760 27356658 PMC5107293

[6] Incecik F Hergüner OM Besen S Yılmaz M Altunbasak S. Limbic encephalitis associated with anti-leucine-rich glioma-inactivated-1 protein antibodies in a child. *Neurol India.* (2016) 64:1321–3. 10.4103/0028-3886.193776 27841212

[7] Schimmel M Frühwald MC Bien CG. Limbic encephalitis with LGI1 antibodies in a 14-year-old boy. *Eur J Paediatr Neurol.* (2018) 22:190–3. 10.1016/j.ejpn.2017.08.004 28919330

[8] Steriade C Day GS Lee L Murray BJ Fritzler MJ Keith J. LGI1 autoantibodies associated with cerebellar degeneration. *Neuropathol Appl Neurobiol.* (2014) 40:645–9. 10.1111/nan.12132 24606111

[9] Irani SR Michell AW Lang B Pettingill P Waters P Johnson MR Faciobrachial dystonic seizures precede Lgi1 antibody limbic encephalitis. *Ann Neurol.* (2011) 69:892–900. 10.1002/ana.22307 21416487

[10] Chen C Wang X Zhang C Cui T Shi WX Guan HZ Seizure semiology in leucine-rich glioma-inactivated protein 1 antibody-associated limbic encephalitis. *Epilepsy Behav.* (2017) 77:90–5. 10.1016/j.yebeh.2017.08.011 29050866

[11] Suleiman J Wright S Gill D Brilot F Waters P Peacock K Autoantibodies to neuronal antigens in children with new-onset seizures classified according to the revised ILAE organization of seizures and epilepsies. *Epilepsia.* (2013) 54:2091–100. 10.1111/epi.12405 24151870

[12] Dubey D Alqallaf A Hays R Freeman M Chen K Ding K Neurological autoantibody prevalence in epilepsy of unknown etiology. *JAMA Neurol.* (2017) 74:397–402. 10.1001/jamaneurol.2016.5429 28166327

[13] Graus F Titulaer MJ Balu R Benseler S Bien CG Cellucci T A clinical approach to diagnosis of autoimmune encephalitis. *Lancet Neurol.* (2016) 15:391–404. 10.1016/S1474-4422(15)00401-9 26906964 PMC5066574

[14] Cellucci T Mater HV Graus F Muscal E Gallentine W Klein-Gitelman MS Clinical approach to the diagnosis of autoimmune encephalitis in the pediatric patient. *Neurol Neuroimmunol Neuroinflamm.* (2020) 7:e663. 10.1212/NXI.0000000000000663 31953309 PMC7051207

